# Insect Stings

**Published:** 1890-08-16

**Authors:** 


### SURGERY.

### INSECT STINGS.

Dr. Philippe Record, of Newark, N.J., has recently written on the advantage of peroxide of hydrogen for the relief of bites from venomous insects. He writes that, while charging his atomizer with peroxide of hydrogen for the treatment of a child with diphtheria, the patient's mother was stung by a hornet. Dr. Record directed the peroxide of hydrogen spray into the wound, which caused all pain to cease, and the swelling rapidly disappeared. Dr. Record believes the poison was thus annihilated, and thinks it quite possible that many other poisons might be destroyed by the same means. This may be very true, but we cannot count upon an atomizer and peroxide of hydrogen being available when required to treat bites and stings of venomous insects. Fortunately this country is tolerably free from such pests. But visitors to the Continent, especially in Italy, and even in Switzerland, not infrequently suffer from bites and stings. There is the mosquito, the sting or proboscis of which insect consists of several lancets which are protruded from a sheath, from which also is ejected an irritating fluid. The result of a bite is a small white hard lump, which, if irritated, may inflame and become a very obstinate sore. This is much more likely to happen if the person bitten is in any way unhealthy; whether the condition is plethoric or anæmic. There are few, if any, veritable mosquitos in this country, although some are occasionally seen. There are, however, some representatives of the genus in the shape of the gnats with which we are familiar in the summer time. It is somewhat curious that although mosquitos are a pest alike in the tropics, and in the Arctic regions, they do not seem able to thrive in the temperate climate. In the absence of peroxide of hydrogen, the best application for a bite is sal volatile, or water may be rubbed well into the part, so that some may enter the wound and dilute the poison.

Wasp and bee stings are common enough in most countries. In Eastern lands bees seem more pugnacious than in temperate climates. For we rarely hear of swarms of bees attacking an individual in England, as they not infrequently do in India. In delicate persons one or two stings may excite constitutional symptoms. When a person is stung severely, there is faintness, nausea, vomiting, and often purging. The stings should be, if possible, extracted by pressure with a key, after which the best application is sal volatile. In cases of stings in the mouth or throat (which may happen when persons bite fruit concealing a wasp) alarming consequences may arise from œdema or swelling, which may extend to the glottis, impeding the entrance of air to the lungs. In the first instance the sting should be extracted if possible, ice should be kept in the mouth, and leeches may be applied externally over the sternum. An emetic has sometimes proved useful, but laryngotomy has been necessitated for the relief of the breathing.

The "bed-bug," *cinex lectularius*, causes much irritation, the place bitten forming a white swelling, which afterwards becomes red. There is no better application than sal volatile. In Eastern countries there are various other kinds of bugs, to which we are strangers. One, called in the native Indian language *jow*, or *jowar*, lives in sandy places, and excites much irritation of the skin. There is also a flying-bug, the scent from which is most disagreeable.

Some spiders are undoubtedly venomous, the poison being elaborated by a gland, and transmitted to the mandibles. It is not, however, known what these species are. Sir J. Fayrer says: "In India a streak of almost erysipelatous redness of the skin is often attributed to a spider." Something of the kind has been observed in the hayfields in this country, when spiders have come into contact with the human skin. Ammonia is the best application.

The scorpion, fortunately, is not found in this country. There are several varieties of scorpions. The tail is the offensive weapon. The scorpion seizes its prey with its palpi, and then pierces it with the sting at the extremity of the tail. The pain is at first like the prick of a needle, but in a few seconds it becomes more acute, as if innumerable pins were being thrust into the part. The pain also shoots towards the body, reaching a climax in about ten minutes. The part injured swells, and frequently the absorbents are implicated, as evidenced by a red line under the skin. The symptoms are more severe if the person is not in good health, and death has been recorded from a scorpion sting. Vinegar, salt and water, sal volatile, are domestic remedies. The best treatment is suction, or the application of cupping glasses, followed by a poultice composed of equal parts of ipecacuanha and powdered opium. If the absorbents inflame they should be fomented. An aperient is advisable.

The centipede also is not met with in this country. It possesses mandibles with an aperture near the apex, through which a poisonous fluid is ejected. The symptoms of centipede bite are very similar, but not so severe, as those of scorpion sting, and the same treatment is desirable.

This does not include the full list of minor venomous injuries, but only those usually presenting. Snake bite cannot be considered under the heading, the danger from such injury removing it from the minor class.

